# Semaglutide vs tirzepatide in patients with obesity and HFpEF: a report from a global federated research network

**DOI:** 10.1093/eschf/xvag042

**Published:** 2026-01-29

**Authors:** Luca Monzo, Gianluigi Savarese, Kevin Duarte, Guillaume Baudry, Mark C Petrie, Nicolas Girerd

**Affiliations:** CHRU de Nancy, Centre d'Investigation Clinique Plurithématique 1433, INSERM, Université de Lorraine, INSERM U1116—DCAC, and F-CRIN INI-CRCT (Cardiovascular and Renal Clinical Trialists), 4 Rue du Morvan, 54511 Vandœuvre-lès-Nancy, France; Department of Clinical Science and Education, Södersjukhuset, Karolinska Institutet, Stockholm, Sweden; CHRU de Nancy, Centre d'Investigation Clinique Plurithématique 1433, INSERM, Université de Lorraine, INSERM U1116—DCAC, and F-CRIN INI-CRCT (Cardiovascular and Renal Clinical Trialists), 4 Rue du Morvan, 54511 Vandœuvre-lès-Nancy, France; CHRU de Nancy, Centre d'Investigation Clinique Plurithématique 1433, INSERM, Université de Lorraine, INSERM U1116—DCAC, and F-CRIN INI-CRCT (Cardiovascular and Renal Clinical Trialists), 4 Rue du Morvan, 54511 Vandœuvre-lès-Nancy, France; School of Cardiovascular and Metabolic Health, University of Glasgow, Glasgow, UK; CHRU de Nancy, Centre d'Investigation Clinique Plurithématique 1433, INSERM, Université de Lorraine, INSERM U1116—DCAC, and F-CRIN INI-CRCT (Cardiovascular and Renal Clinical Trialists), 4 Rue du Morvan, 54511 Vandœuvre-lès-Nancy, France

**Keywords:** Heart failure, Semaglutide, Tirzepatide, Obesity

## Abstract

**Background and Aims:**

Semaglutide and tirzepatide have been shown to reduce body weight, improve health status, and lower rates of clinical events in patients with obesity and heart failure with preserved ejection fraction (HFpEF). Although recent data suggest that tirzepatide leads to greater weight loss compared to semaglutide in non-HF populations, it remains uncertain whether these different drugs might result in different clinical event rates. This study aims to compare the rates of clinical outcomes for semaglutide vs tirzepatide in patients with obesity and HFpEF.

**Methods:**

In this non-randomized, observational cohort study, adults with obesity and a concurrent diagnosis of HFpEF who initiated treatment with semaglutide or tirzepatide for the first time between November 2023 and May 2025 were identified using electronic health record data from the TriNetX Global Collaborative Research Network. The primary endpoint was a composite of all-cause mortality and HF hospitalization, evaluated after propensity score matching (PSM).

**Results:**

Among 3983 patients meeting the study criteria (semaglutide, 2719; tirzepatide, 1264), 1258 remained in each group after PSM (mean age 66 years, 41% male, 77% White, mean body mass index 42 kg/m², 63% with diabetes). Over a median follow-up of 24 weeks, semaglutide and tirzepatide were associated with a similar risk of the primary composite endpoint (HR 1.14 [95% CI, 0.89–1.46]; *P* = .286), and of its individual components (all-cause death: HR 1.24 [95% CI, 0.63–2.44]; *P* = .531; HF hospitalization: HR 1.10 [95% CI, 0.85–1.43]; *P* = .471), irrespective of diabetes status.

**Conclusions:**

In this real-world analysis, no difference was observed between semaglutide and tirzepatide in terms of clinical outcomes among patients with obesity and HFpEF.

## Graphical Abstract

**Graphical Abstract:**
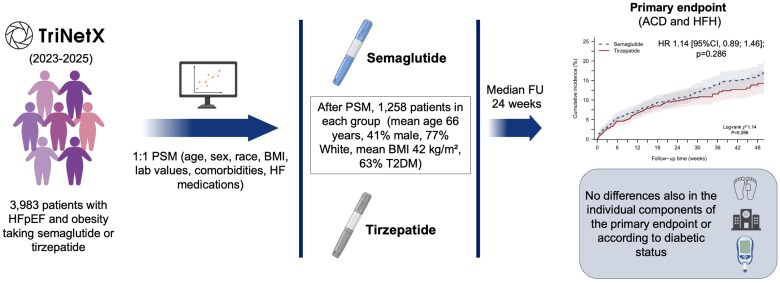
Study design and key findings of the study. Patients were selected from the TriNetX network between 2023 and 2025, including individuals with obesity and HFpEF receiving treatment with either semaglutide or tirzepatide (left). A 1:1 propensity score–matched design was applied to balance demographic characteristics, BMI, laboratory values, comorbidities, and HF medications between treatment groups (middle). Over a median follow-up of 24 weeks, semaglutide and tirzepatide showed similar risks for the composite endpoint of all-cause death and HF hospitalization, with no differences in individual components or by diabetic status (right). Abbreviations: ACD, all-cause death; HFH, heart failure hospitalization; HFpEF, heart failure with preserved ejection fraction; PSM, propensity score matching; T2DM, type 2 diabetes mellitus.

## Introduction

The glucagon-like peptide-1 receptor agonist (GLP-1 RA) semaglutide and the dual GLP-1 RA/gastric inhibitory polypeptide (GIP) receptor agonist tirzepatide have demonstrated significant weight reduction in patients with obesity and heart failure with preserved ejection fraction (HFpEF), along with improvements in health status.^1^ In three moderately sized randomized trials, semaglutide and tirzepatide were associated with lower rates of HF events compared to placebo in patients with obesity-related HFpEF.^2–4^ However, the limited number of events in these studies precludes definitive conclusions. Notably, while recent data indicate that tirzepatide induces greater weight loss than semaglutide in individuals with overweight or obesity without HFpEF,^5,6^ it remains unclear whether this is the case in HFpEF or if the different drug classes are associated with differential HF event rates.

We compared HF event rates for semaglutide (injectable) vs tirzepatide on mortality and HF hospitalizations using real-world data from a large, global federated electronic health record (EHR) database.

## Methods

### Study design and data derivation

We conducted a retrospective analysis using the TriNetX Global Collaborative Research Network, a global platform encompassing more than 275 million patients worldwide that provides real-world longitudinal clinical data, including demographics, diagnoses, procedures, laboratory results, and medication prescriptions sourced from EHRs and pharmacy claims across academic and community-based institutions. At the time the query was executed, the Global Collaborative Network comprised 172 participating healthcare organizations across 19 countries, representing approximately 170 million patients.

Participating healthcare organizations contribute de-identified, aggregated datasets mapped to a master clinical terminology aligned with standardized coding ontologies, such as the 10th edition of the International Classification of Diseases (ICD-10), Current Procedural Terminology (CPT), Logical Observation Identifiers Names and Codes (LOINC), RxNorm, TriNetX (TNX), curated, and Anatomical Therapeutic Chemical (ATC) codes.^7^ Notably, the identification of medical conditions, such as HFpEF and diabetes mellitus, was based on ICD-10 codes recorded in EHRs. Rigorous quality controls, such as validation against clinical standards and expert review, ensure data reliability.^8^ TriNetX performs rigorous data preprocessing to minimize missing values, mapping data to a consistent clinical model that supports uniform querying across diverse sources. Covariates are standardized as binary, categorical (expanded into binary columns), or continuous. Age is always available, and missing values for sex, race, and ethnicity are represented as unknown’. Given the use of only aggregate de-identified data, this study was exempted from Institutional Review Board approval. The study adhered to STROBE (STrengthening the Reporting of OBservational studies in Epidemiology) guidelines for observational research.

### Study population

The observation period extended from November 2023, the date tirzepatide was approved in the USA for the treatment of obesity, to May 2025, to ensure both treatment groups were evaluated over the same follow-up duration and within a contemporaneous population, given that semaglutide had been approved earlier (June 2021). Over this timeframe, we identified 3983 patients with obesity (body mass index ≥30) who were prescribed semaglutide (injectable, not oral; *n* = 2719) or tirzepatide (*n* = 1264) for the first time, and who had a concurrent diagnosis of HFpEF (ICD-10: I50.3) within 6 months of the prescription, with or without type II diabetes (ICD-10: E11) (data accessed on 21 May 2025). The codes used to select the population are listed in Supplementary Table S1.

### Statistical analysis

Statistical analyses were conducted on the TriNetX online research platform, which enables patient-level analyses while only reporting population-level data through its built-in analytical functions (i.e. incidence, prevalence, outcomes, survival analysis, propensity score matching).

Propensity score matching (PSM) was performed on TriNetX platform with 1:1 matching using logistic regression for semaglutide group vs tirzepatide group, employing nearest-neighbour matching with a tolerance of 0.01 and PSM calliper of 0.1 pooled standard deviations. Matching variables were selected based on their potential impact on outcomes and included demographics (age, sex, race [White, Black or African American, Asian, Other, Unknown], ethnicity [Hispanic or Latino, Not Hispanic or Latino, Unknown]), anthropometrics (body mass index), laboratory values (haemoglobin, creatinine, glucose, natriuretic peptides), medical treatment (betablockers, renin-angiotensin-aldosterone system inhibitors, loop diuretics, sodium-glucose contransporter-2 inhibitors [SGLT2i], oral antidiabetic drugs [metformin, sulfonylureas, dipeptidyl peptidase 4 inhibitors], insulin, aldosterone antagonist [MRA]) and relevant health conditions identified from EHRs (hypertensive disease, ischaemic heart disease, diabetes mellitus, atrial fibrillation/flutter), as done previously (Supplementary Table S2).^9,10^ The standardized mean difference (SMD) was used to quantify the differences between the two groups, both before and after statistical weighting. Missing values were not imputed and instead categorized as ‘unknown’. When PSM was applied, patients with missing values in matching variables were excluded from the matched cohorts.

The primary outcome was a composite of all-cause mortality and HF hospitalization, starting from semaglutide or tirzepatide treatment initiation (Supplementary Table S3). Hazard ratios (HR) with 95% confidence intervals (CI) were calculated to compare the time-to-event outcome. Kaplan–Meier curves and log-rank tests were used to illustrate survival analysis. Patients were right-censored at the time of their last available clinical record in TriNetX if this occurred within the prespecified observation window. For each Cox analysis performed, proportional hazards assumptions were assessed using platform-provided tests, with no evidence of meaningful deviation. Statistical significance was set at a two-sided alpha <0.05.

## Results

### Baseline characteristics

Before PSM, the two groups were quite similar, although the semaglutide group showed a slightly higher prevalence of comorbidities (hypertension, diabetes, ischaemic heart disease, and atrial fibrillation), along with modestly higher BMI and blood glucose levels. These minor imbalances were fully corrected after matching, resulting in two groups of 1258 patients each with no differences in baseline characteristics (*Table 1*; Supplementary Figure S1).

**Table 1:** Baseline clinical characteristics of the semaglutide cohort and tirzepatide cohort before and after statistical weighting

	Before PSM			After PSM		
	Semaglutide (*n* = 2719)	Tirzepatide (*n* = 1264)	SMD	Semaglutide (*n* = 1258)	Tirzepatide (*n* = 1258)	SMD
Demographics						
Age, years	65.9 ± 11.4	65.6 ± 11.8	.024	65.8 ± 11.5	65.7 ± 11.7	.016
Male sex	41	40	.017	41	40	.019
BMI, kg/m^2^	41.1 ± 7.9	41.8 ± 8.1	.086	41.5 ± 7.9	41.8 ± 8.1	.037
Weight, kg	115 ± 27	117 ± 26	.081	117 ± 28	117 ± 26	.035
Race						
White	74.8	77.2	.056	77.1	77.2	.002
Black or African American	15.8	15.2	.017	14.4	15.2	.022
Asian	1.4	.9	.039	1.0	1.0	.008
Other	3.4	2.3	.066	2.3	2.2	.007
Unknown	4.6	4.4	.101	5.2	4.4	.037
Ethnicity						
Not Hispanic or Latino	71.1	74.5	.077	75.7	74.4	.029
Hispanic or Latino	4.9	4.4	.027	4.8	4.4	.019
Unknown	24.0	21.1	.068	19.5	21.2	.041
Comorbidities						
Hypertension	95.3	93.3	.087	93.3	93.7	.016
Ischemic heart disease	60.9	57.4	.071	57.1	57.5	.009
Diabetes mellitus	66.7	63.7	.064	63.4	63.8	.008
Atrial fibrillation or flutter	35.0	32.1	.060	34.3	32.3	.041
Blood						
Creatinine, mg/dl	1.2 ± 1.3	1.3 ± 3.0	.049	1.2 ± .9	1.3 ± 3.0	.058
Glucose, mg/dl	134 ± 57	127 ± 51	.121	130 ± 54	127 ± 52	.056
Haemoglobin, gr/l	13.0 ± 2.0	13.0 ± 2.0	.014	13.1 ± 2.0	13.0 ± 2.0	.004
HbA1c	6.8 ± 1.6	6.7 ± 1.6	.073	6.7 ± 1.5	6.7 ± 1.6	.018
Medical treatment						
Sacubitril/valsartan	4.4	4.2	.009	4.8	4.2	.031
ACE inhibitors	44.4	40.7	.073	41.7	40.9	.015
ARBs	48.3	47.8	.010	47.8	48.0	.005
MRA	31.8	33.1	.029	34.2	33.1	.022
SGLT2 inhibitors	36.7	35.4	.028	35.2	35.3	.002
Betablockers	80.3	78.6	.042	78.9	78.8	.002
Loop diuretics	80.0	78.6	.034	78.9	78.9	<.001
OAD						
Metformin	40.4	37.4	.062	37.3	37.4	.003
Sulfonylureas	15.4	13.8	.047	14.6	13.8	.023
DPP-4 inhibitors	9.5	7.8	.060	8.7	7.9	.032
Insulin	49.9	47.8	.070	45.9	46.4	.009

ACE, angiotensin converting enzyme; ARBs, angiotensin receptor blockers; BMI, body mass index; DPP-4, dipeptidyl peptidase 4; HbA1c, glycated haemoglobin; MRA, mineralocorticoid receptor antagonist; PSM, propensity score matching; SGLT2, sodium-glucose co-transporter-2; OAD, oral antidiabetic drugs; SMD, standardized mean difference.

Values are proportion (%) or mean ± standard deviation

### Clinical outcomes

**Figure 1:**
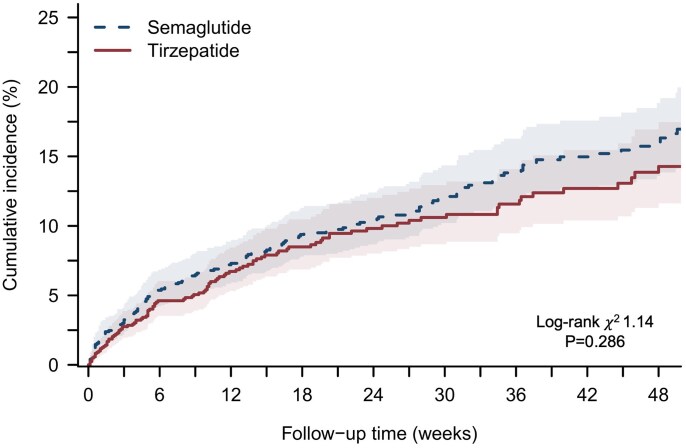
Kaplan–Meier curves for the composite outcome of all-cause death and heart failure hospitalization in patients with heart failure with preserved ejection fraction and obesity treated with semaglutide or tirzepatide

Over a median follow-up of 24 weeks (IQR 34) in the pooled population, there was no difference between the two groups in terms of the primary composite endpoint of all-cause mortality and HF hospitalization in patients with HFpEF and obesity (semaglutide 151 events [12.0%] vs tirzepatide 112 events [8.9%]; HR 1.14, [95% CI, 0.89–1.46]; *P* = .286) (*Figure 1*). Similarly, no differences between the two groups were observed for the individual components of the composite endpoint (all-cause death: HR 1.24 [95% CI, 0.63–2.44]; *P* = .531; HF hospitalization: HR 1.10 [95% CI: 0.85–1.43] *P* = .471).

Subgroup analyses also showed no difference between the two treatments in patients with (*N* = 805 per group after PSM; HR 1.04, [95% CI 0.78–1.40]; *P* = .781) or without diabetes (*N* = 431 per group after PSM; HR 1.02, [95% CI 0.62–1.66]; *P* = .939) (Supplementary Figure S2).

## Discussion

In this real-world, EHR-based cohort study of adults with HFpEF and obesity who initiated treatment with semaglutide or tirzepatide, we observed no difference in the risk of all-cause mortality or HF hospitalization, irrespective of diabetes status (*Graphical Abstract*).

Recent observations have demonstrated greater weight reduction with tirzepatide compared to semaglutide in non-HF populations. In a large EHR-based cohort study of 18 386 US adults with overweight or obesity, tirzepatide use was associated with greater weight loss than semaglutide at multiple time points (−2.4% at 3 months, −4.3% at 6 months, and −6.9% at 12 months).^5^ Similarly, the SURMOUNT-5 trial (A Study of Tirzepatide in Participants With Obesity or Overweight With Weight-Related Comorbidities) demonstrated that once-weekly administration of tirzepatide at the maximum tolerated dose (10 or 15 mg) resulted in greater reductions in body weight (−6.5%, [95% CI −8.1% to −4.9%]) and waist circumference (−5.4 cm, [95% CI −7.1 to −3.6]) compared with semaglutide (1.7 or 2.4 mg) in non-diabetic patients with obesity.^6^

A slightly greater effect on weight reduction with tirzepatide compared to semaglutide was also observed in HFpEF obesity trials. In the STEP-HFpEF (Effect of Semaglutide 2.4 mg Once Weekly on Function and Symptoms in Subjects With Obesity-Related Heart Failure With Preserved Ejection Fraction) trial and in the STEP-HFpEF DM trial (Semaglutide Treatment Effect in People with Obesity and Heart Failure with Preserved Ejection Fraction and Diabetes Mellitus) trial, semaglutide 2.4 mg once weekly resulted in a mean in body weight reduction of 10.7% and 9.8%, respectively, at 52 weeks.^2,3^ In comparison, the SUMMIT (Tirzepatide for Heart Failure With Preserved Ejection Fraction and Obesity) trial reported an 11.6% weight reduction at 52 weeks with tirzepatide, administered up to 15 mg weekly.^4^ Both trials showed potentially favourable effects on clinical outcomes. Semaglutide demonstrated a signal of benefit in lowering HF events (HR 0.08, based on 13 events, 1 in semaglutide group), while tirzepatide reduced the composite of cardiovascular death (13 events, 8 in the tirzepatide group) or worsening HF (81 events, 29 in the tirzepatide group) by nearly 40%, primarily through a reduction in worsening HF events (HR 0.54, [95%CI 0.34–0.85]), with no apparent effect on all-cause mortality. Notably, in these trials, the effects of GLP-1 receptor agonists on symptoms, functional capacity, and haemodynamics appeared largely independent of glycaemic status. This is consistent with our real-world findings, in which no evidence of effect modification by diabetes was observed. This pattern is biologically plausible, as GLP-1-based therapies exert effects beyond glucose lowering, including weight reduction, anti-inflammatory actions, and haemodynamic modulation, which target key pathophysiological mechanisms of obesity-related HFpEF shared by patients with and without diabetes.^1,11^

In a recent meta-analysis of 3743 HFpEF patients from four randomized, placebo-controlled trials (SELECT, FLOW, STEP-HFpEF, and STEP-HFpEF DM), semaglutide significantly reduced the risk of worsening HF events (54 [2.8%] vs 86 [4.7%]; HR 0.59 [95% CI, 0.41–0.82]), while its effect on cardiovascular death alone was not significant.^12^ However, given the differences in study design and endpoints, it remains unclear whether the greater weight reduction observed with tirzepatide or the non-weight-dependent effects of these incretin therapies translate into differential rates of clinical outcomes compared to semaglutide. Our analysis provides an initial, though not exhaustive, response to this question, showing similar mortality and HF event rates. Semaglutide might differ from tirzepatide in terms of HF events because of tirzepatide’s role as a GIP receptor agonist. This mechanism may account for the additional weight loss observed with tirzepatide, which does not necessarily translate into further benefits on cardiovascular outcomes. Other non-weight loss mechanisms (i.e. enhanced glucose-dependent insulin secretion and insulin sensitivity, decreased energy consumption) are likely to contribute to the observed effects.^13^ Indeed, improvements in health-related domains seen with tirzepatide in patients with obesity and HFpEF^4^ appear comparable to those observed with semaglutide in the STEP-HFpEF trial, particularly with respect to functional status, symptom burden, six-minute walk distance,^2,14^ and reduction in diuretic dose.^15^ In this context, although GLP-1-based therapies induce early metabolic and haemodynamic effects, potential structural cardiac changes may require more sustained exposure, with corresponding effects on clinical outcomes emerging later.^11^ Nevertheless, available real-world evidence suggests that even with longer follow-up (up to 52 weeks) no meaningful differences in mortality or HF hospitalization emerged between tirzepatide and semaglutide.^16^ Moreover, although follow-up in our study was shorter, the observed event rate was higher (∼9%–12% at 6 months vs ∼3.5% at 52 weeks), which may have increased the likelihood of detecting clinically relevant differences, if present.

Our study is subject to several limitations. As a retrospective observational analysis, causal inferences cannot be made. We cannot exclude the possibility that a longer observation period might have revealed differences in event rates between the two drugs. However, as discussed above, given the event burden observed in our cohort and the absence of meaningful differences in clinical outcomes between semaglutide and tirzepatide in longer real-world head-to-head analyses,^16^ it appears unlikely that limited follow-up alone fully explains the neutral findings. Patients in the TriNetX database represent individuals with healthcare encounters within participating systems and may not reflect the general population. The study is also subject to common limitations of EHR-based observational research, including diagnostic inaccuracies, unmeasured confounding, selection bias, and reverse causality. In particular, the identification of HFpEF based on ICD-10 diagnostic codes may be associated with some degree of misclassification. Although background HF therapies were well balanced between treatment groups, the overall use of SGLT2i and MRAs was relatively low, likely reflecting real-world prescribing patterns and a period of early guideline implementation for SGLT2i, which may limit the generalizability of our findings. All analyses were conducted using the built-in TriNetX analytics platform, which applies a standardized logistic regression model for propensity score estimation. As a result, additional sensitivity analyses (i.e. inverse probability weighting, multivariable Cox regression) were not feasible due to platform-related technical constraints. Despite this limitation, the matched cohorts demonstrated excellent covariate balance, supporting adequate adjustment for the available covariates. In addition, the TriNetX platform does not provide access to individual-level time-to-event data, precluding the derivation of numbers at risk at specific time points and limiting more granular survival analyses. We were unable to directly assess the severity of HF and its comorbidities, which may have introduced potential bias into our findings. EHR-derived medication data did not allow detailed assessment of treatment indication (HFpEF- vs diabetes-labelled use), dosing, treatment persistence, or adherence over time. However, as this study restricted treatment initiation to prescriptions occurring within 6 months of HFpEF diagnosis, this temporal constraint makes such an indication more likely, although it cannot be formally verified. In addition, treatment discontinuation or modification for reasons not captured in the database, such as financial burden, adverse effects, or patient preference, may have occurred. Finally, longitudinal data on body weight or weight change during follow-up were not available, precluding assessment of differential weight trajectories between semaglutide and tirzepatide and their potential relationship with clinical outcomes.

In conclusion, this real-world analysis from a global federated research dataset found no difference between semaglutide and tirzepatide in terms of rates of adverse clinical outcomes among patients with HFpEF and obesity. In this setting, further adequately powered randomized trials are needed to definitively assess potential differences in treatment effects between these drugs.

## Supplementary Material

xvag042_Supplementary_Data

## Declarations

### Data Availability

All data used in the generation of this paper were collected from the global TriNetX network. The data that support the findings of this study are available from TriNetX, LLC, https://trinetx.com/, but third-party restrictions apply to the availability of these data. The data were used under license for this study with restrictions that do not allow for the data to be redistributed or made publicly available. However, for accredited researchers, the TriNetX data are available for licensing at TriNetX, LLC. Data access may require a data sharing agreement and may incur data access fees.
