# Supplementary material for: Semaglutide vs tirzepatide in patients with obesity and HFpEF: a report from a global federated research network
Source: ESC Heart Fail. 2026 Jan 29;13(1):xvag042. doi: 10.1093/eschf/xvag042 (PMC13108322; doi:10.1093/eschf/xvag042)

**Supplemental Table 1** The 10^th^ edition of the International Classification of Diseases (ICD-10), Logical Observation Identifiers Names and Codes (LOINC), TriNetX (TNX) Curated, and RxNorm codes used to select the study population

| **Code** | **Definition** |
| --- | --- |
| **ICD-10** | |
| I50 | Heart failure |
| I50.1 | Left ventricular failure, unspecified |
| I50.2 | Systolic (congestive) heart failure |
| I50.3 | Diastolic (congestive) heart failure |
| I50.4 | Combined systolic (congestive) and diastolic (congestive) heart failure |
| E11 | Type 2 diabetes mellitus |
| Z68.3 | Body mass index [BMI] 30-39, adult |
| Z68.4 | Body mass index [BMI] 40 or greater, adult |
| **LOINC** | |
| 39156-5 | Body mass index (>30 kg/m^2^) |
| **TNX curated** |  |
| 9083 | BMI (>30 kg/m^2^) |
| **RxNorm** | |
| 26011723 | Tirzepatide |
| 1991302 | Semaglutide |
| 1440051 | Lixisenatide |
| 1551291 | Dulaglutide |
| 475968 | Liraglutide |
| 60548 | Exenatide |

**Supplemental Table 2** Covariates used in the propensity score matching process and relative coding

| **Covariates** | **Code** | **Definition** |
| --- | --- | --- |
| **Demographics** |  |  |
| Age | - | - |
| Sex | - | Male |
| Race | - | White |
|  | - | Black or African American |
|  | - | Asian |
|  | - | Other |
| Ethnicity | - | Not Hispanic or Latino |
|  | - | Hispanic or Latino |
| **Diagnosis (ICD-10 coding)** | | |
|  | I10-I1A | Hypertensive disease |
|  | I20-I25 | Ischemic heart diseases |
|  | E08-E13 | Diabetes mellitus |
| **Medications (VA coding)** |  |  |
|  | CV100 | Beta Blockers/Related |
|  | CV702 | Loop Diuretics |
|  | CV800 | ACE Inhibitors |
|  | CV805 | Angiotensin II Inhibitors |
|  | HS501 | Insulin |
| **Medications (ATC coding)** |  |  |
|  | C03DA | Aldosterone antagonists (MRAs) |
|  | A10BK | Sodium-glucose co-transporter 2 (sglt2) inhibitors |
|  | A10BA | Biguanides |
|  | A10BB | Sulfonylureas |
|  | A10BH | Dipeptidyl peptidase 4 (dpp-4) inhibitors |
| **Medications (Rxnorm coding)** |  |  |
|  | 1656328 | Sacubitril/Valsartan |
| **Laboratory (TNX curated coding)** | | |
|  | 9024 | Creatinine [Mass/volume] in Serum, Plasma or Blood |
|  | 9083 | Body mass index |
|  | 9014 | Haemoglobin [Mass/volume] in Blood |
|  | 9072 | Natriuretic peptide B prohormone N-Terminal [Mass/volume] in Serum, Plasma or Blood |
|  | 9003 | Natriuretic peptide B [Mass/volume] in Serum, Plasma or Blood |

**Supplemental Table 3** The 10^th^ edition of the International Classification of Diseases (ICD-10) codes used for outcomes definition

| **Outcome** | **ICD-10 code** | **Definition** |
| --- | --- | --- |
| All cause death | - | Deceased |
| Heart failure hospitalization | I50.21 | Acute systolic (congestive) heart failure |
|  | I50.31 | Acute diastolic (congestive) heart failure |
|  | I50.33 | Acute on chronic diastolic (congestive) heart failure |
|  | I50.23 | Acute on chronic systolic (congestive) heart failure |
|  | I50.43 | Acute on chronic combined systolic (congestive) and diastolic (congestive) heart failure |
|  | I50.41 | Acute combined systolic (congestive) and diastolic (congestive) heart failure |

**Supplemental Figure 1** Love plot showing standardized mean differences of baseline covariates before (red circles) and after (blue squares) propensity score matching. Dashed vertical line represents the prespecified threshold for acceptable balance. Abbreviations: ACE, angiotensin converting enzyme; AF, atrial fibrillation; BMI, body mass index; BNP, brain natriuretic peptide; DM, diabetes mellitus; DPP-4, dipeptidyl peptidase 4; HF, heart failure; NT-proBNP, N-terminal pro–brain natriuretic peptide; SGLT2i, sodium-glucose co-transporter 2 inhibitor.

**
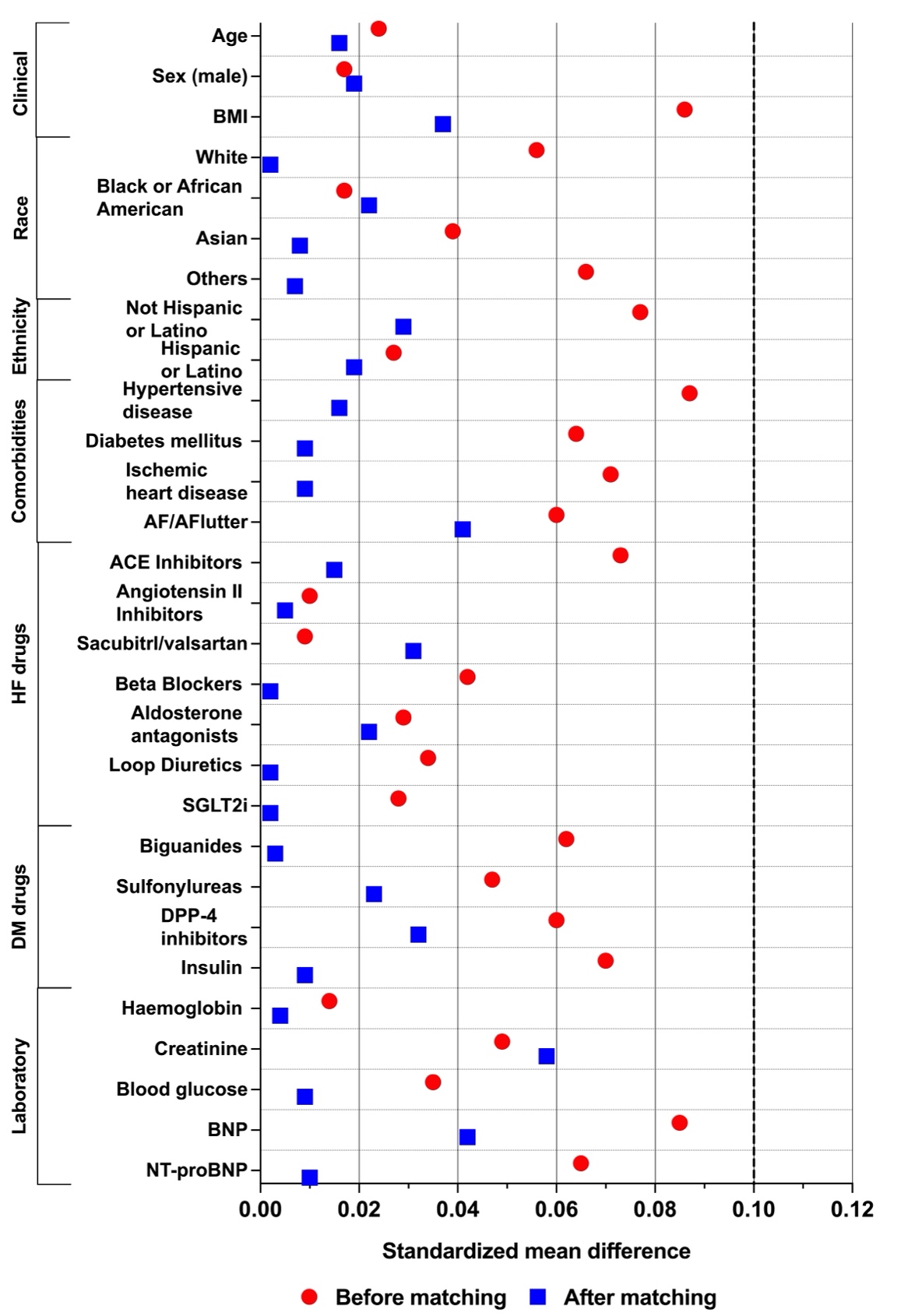
**

**Supplemental Figure 2** Kaplan–Meier curves for the composite outcome of all-cause death and heart failure hospitalization in **A)** diabetic and **B)** non-diabetic patients with heart failure with preserved ejection fraction and obesity treated with semaglutide or tirzepatide.


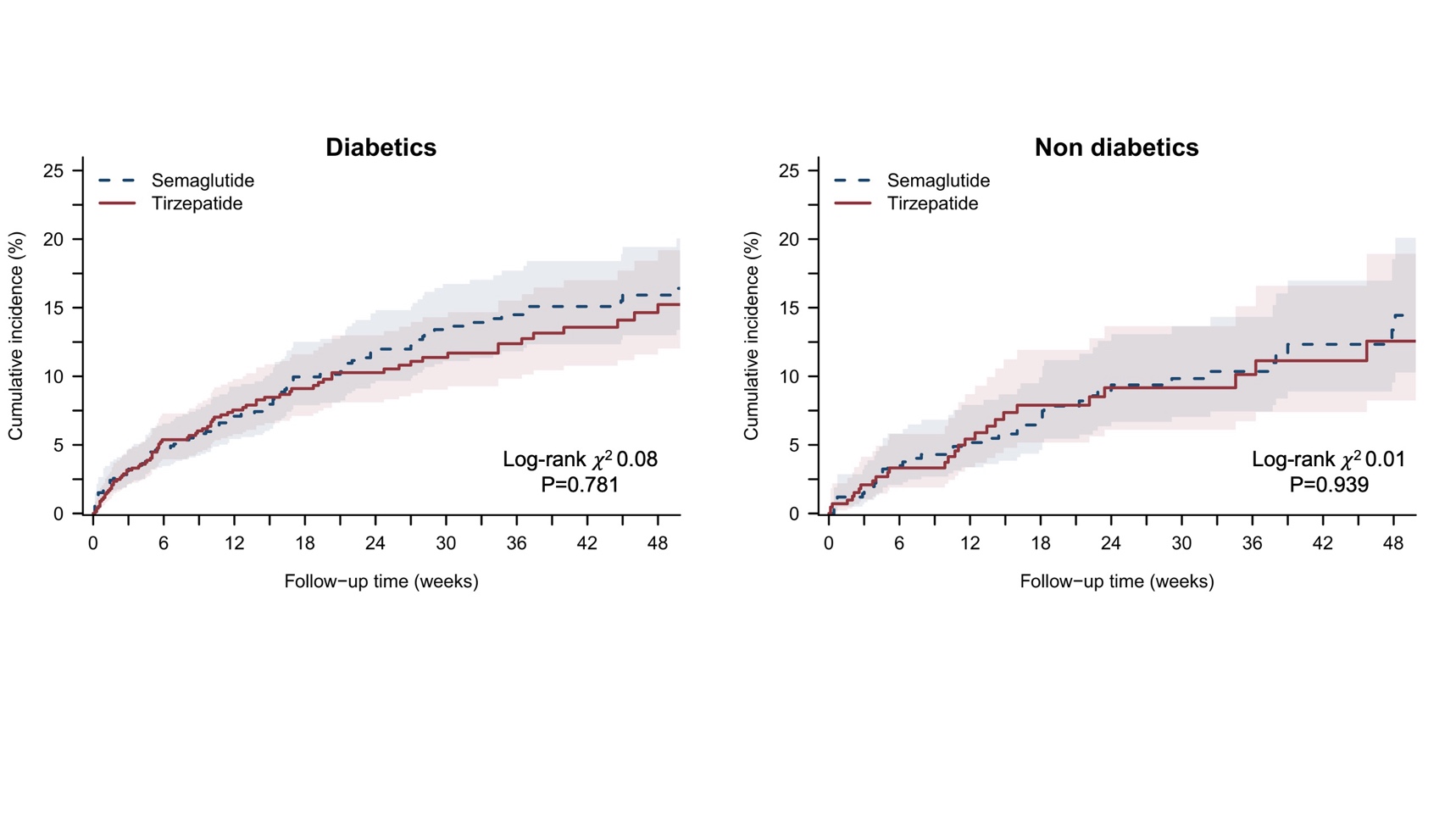

Supplement: xvag042_Supplementary_Data [file xvag042_supplementary_data.docx]
